# Posttranslational regulation of Fas ligand function

**DOI:** 10.1186/1478-811X-6-11

**Published:** 2008-12-29

**Authors:** Matthias Voss, Marcus Lettau, Maren Paulsen, Ottmar Janssen

**Affiliations:** 1 Molecular Immunology, Institute of Immunology, Medical Center Schleswig-Holstein Campus Kiel, Arnold-Heller-Str. 3, Bldg. 17, D-24105 Kiel, Germany

## Abstract

The TNF superfamily member Fas ligand acts as a prototypic death factor. Due to its ability to induce apoptosis in Fas (APO-1, CD95) expressing cells, Fas ligand participates in essential effector functions of the immune system. It is involved in natural killer cell- and T cell-mediated cytotoxicity, the establishment of immune privilege, and in termination of immune responses by induction of activation-induced cell death. In addition, Fas ligand-positive tumours may evade immune surveillance by killing Fas-positive tumour-infiltrating cells. Given these strong cytotoxic capabilities of Fas ligand, it is obvious that its function has to be strictly regulated to avoid uncontrolled damage. In hematopoietic cells, the death factor is stored in secretory lysosomes and is mobilised to the immunological synapse only upon activation. The selective sorting to and the release from this specific lysosomal compartment requires interactions of the Fas ligand cytosolic moiety, which mediates binding to various adapter proteins involved in trafficking and cytoskeletal reorganisation. In addition, Fas ligand surface expression is further regulated by posttranslational ectodomain shedding and subsequent regulated intramembrane proteolysis, releasing a soluble ectodomain cytokine into the extracellular space and an N-terminal fragment with a potential role in intracellular signalling processes. Moreover, other posttranslational modifications of the cytosolic domain, including phosphorylation and ubiquitylation, have been described to affect various aspects of Fas ligand biology. Since FasL is regarded as a potential target for immunotherapy, the further characterisation of its biological regulation and function will be of great importance for the development and evaluation of future therapeutic strategies.

## Background

Fas ligand (FasL, APO-1L, CD95L, CD178) belongs to the tumour necrosis factor (TNF) superfamily of type II transmembrane proteins [1]. It was initially cloned from a T cell hybridoma that strongly bound murine Fas-Fc fusion proteins [2]. Binding of membrane-bound FasL to Fas/APO-1/CD95-expressing target cells triggers a well-characterised pro-apoptotic signalling cascade eventually leading to caspase activation and cell death [3,4]. As a potent death factor, FasL is employed by cytotoxic T lymphocytes (CTLs) and natural killer (NK) cells to selectively kill virus infected or tumourigenic cells and thus complements the perforin/granzyme-dependent effector pathway [5]. Of note, however, beside these two generally accepted key pathways, recent evidence also suggested a significant contribution of TNF-related apoptosis-inducing ligand (TRAIL) in target cell destruction, for example during CTL-mediated clearance of influenza virus infected cells [6].

Apart from its involvement in CTL- and NK cell-mediated cell death, the FasL/Fas system is of particular importance for the immune homeostasis. Its role in down-regulation of immune responses during activation-induced cell death (AICD) is well documented [3,7]. It is underscored by the pathologic phenotypes of naturally occurring Fas- and FasL-deficient mice (*lpr/lpr *and *gld/gld*, respectively), which are defective in AICD and suffer from lymphoproliferative syndromes [8]. Of course, AICD is a complex process that besides the involvement of the FasL/Fas system is further controlled by a number of activation-dependent cytokines, intracellular pro- or anti-apoptotic regulators such as for example Bcl-2 family proteins and signalling molecules like the hematopoietic progenitor kinase (HPK) [9].

Although still a matter of debate with regard to its physiological relevance, FasL expression has been observed in immune-privileged tissues such as the anterior chamber of the eye, as well as in neurons and astrocytes of the central nervous system. It was postulated that in such areas or cells, the death factor might contribute to the protection from inflammation [10]. Interestingly, FasL expression was also observed in tumour cells of non-lymphoid origin and it was speculated that establishment of a tumour-associated immune privilege might enable malignantly transformed cells to evade immune surveillance and possibly also to selectively kill infiltrating lymphocytes [11]. This "tumour counterattack", however, is also still a matter of controversy, since *in vivo*, tumour-associated FasL expression might rather yield pro-inflammatory effects [12]. This goes in line with accumulating evidence for various non-apoptotic signalling processes induced by several TNF family members, and also by FasL binding to Fas [13]. As a consequence, it was recently suggested that Fas (CD95) signalling in general has to be reconsidered since apoptotic versus pro-inflammatory effects strongly depend on the cellular microenvironment [14].

In contrast to the Fas receptor which is expressed in a variety of cell types [15], FasL expression is much more restricted. It is constitutively or inducibly expressed in hematopoietic cells, particularly in NK cells, CTLs and also CD4^+ ^T helper 1 (Th1) cells [16,17]. Due to its non-apoptotic signalling capacity and its neuronal expression, however, FasL-Fas signalling also emerged as potential regulator of neuroplasticity [18].

**Figure 1:**
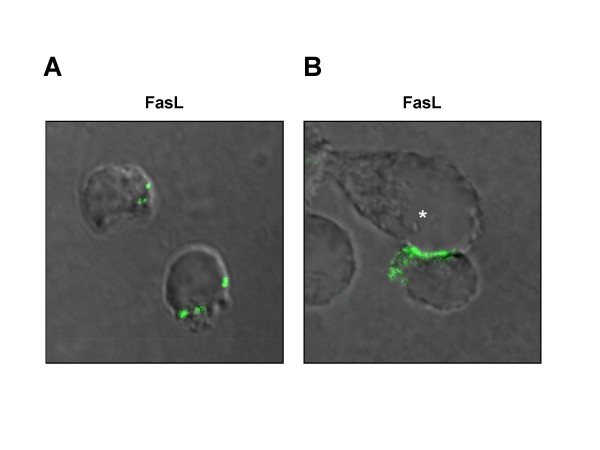
**Localisation of endogenous FasL in primary human T cells**. In human T cell blasts, FasL is stored within the cell in distinct intracellular granules (A). Upon target cell encounter, these granules are transported to the site of intercellular contact, thus placing FasL on the cell surface in the immunological synapse (B). T cell blasts were left untreated (A) or incubated with a superantigen-pulsed EBV-transformed B-cell lymphoblastic cell line (*) for 30 minutes (B), fixed, permeabilised and stained for endogenous FasL with anti-FasL mAb NOK-1 and AlexaFluor 488-conjugated donkey anti-mouse IgG.

Given its strong death-inducing activity, FasL is a potentially dangerous protein and its surface expression has to be strictly regulated in order to prevent unwanted damage. On the one hand, this can be attributed to a complex transcriptional regulation by a number of transcription factors, including NFAT, NF-κB, myc, IRF-1, and others, which has been reviewed elsewhere [7,19]. Often, however, FasL surface expression has to be rapidly induced in an activation- or target cell-restricted fashion. As we know now, this rapid mobilisation, on the other hand, is widely dependent on various posttranslational modification events. In this context, we and others described a biphasic surface expression pattern of FasL in cytotoxic cells [20,21]. Prior to the target cell contact, FasL is retained in mature secretory lysosomes, presumably by protein-protein interactions of its proline-rich domain (PRD) [22]. Only upon activation, for example by T cell receptor (TCR) engagement, these secretory lysosomes are rapidly transported and released into the cytotoxic immunological synapse (Fig. 1). A later increase in surface FasL then relies on its *de novo *transcription and translation.

Only recently, several other potentially important observations have been made regarding the biology of TNF family members and especially FasL. We identified ADAM10 as FasL sheddase and thereby showed that posttranslational regulation of FasL also includes the release of a soluble FasL cytokine from cell surfaces by ectodomain shedding [23]. This finding was meanwhile confirmed by several groups and it was shown that a further intramembrane proteolysis performed by a γ-secretase-like enzyme would release N-terminal fragments with potential intracellular signalling functions [24]. Moreover, as for several other TNF family members [25], recent studies provided evidence for additional mechanisms that contribute to the overall FasL reverse signalling capacity [26]. Thus, phosphorylation of intracellular tyrosine and serine residues or constitutive and inducible protein-protein interactions have been implicated in the initiation or modification of various signalling pathways.

This review focuses on how posttranslational modifications of a crucial death factor relate to biological functions. In this respect, the FasL might be regarded as an example for complexity. This is also reflected by the large cohort of putative interactors for its cytosolic region and the number of irreversible and reversible alterations that regulate FasL expression and intracellular function.

### FasL structure and posttranslational modifications

**Figure 2:**
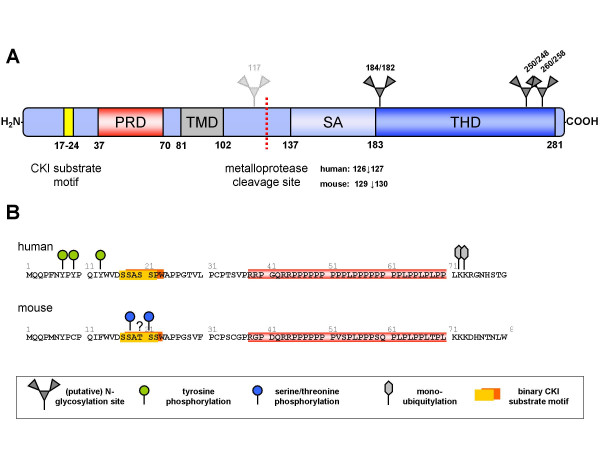
**FasL structure and potential sites of posttranslational modifications**. Schematic representation of FasL structure (A). FasL is a type II membrane protein characterised by its unique cytosolic tail containing a binary casein kinase I (CKI) substrate motif (yellow) and a proline-rich domain (PRD). C-terminal to its transmembrane region (TM), a stretch comprising aa 137 to 183 in human FasL was shown to be essential for trimerisation and self-assembly of FasL (SA). The C-terminal moiety is characterised by its homology to other TNF ligand ectodomains (THD). Cleavage sites for metalloprotease-mediated ectodomain release have been mapped for human FasL in a human and murine cellular environment as indicated. FasL contains three putative glycosylation sites (N184, N250, N260), whereas murine FasL contains four (N117, N182, N248, N258). Previously described covalent posttranslational modifications of the human FasL and murine FasL cytosolic tails (B). As detailed in our review, murine FasL might be subject to serine/threonine phosphorylation of the CKI substrate region, but such sites have not been exactly mapped to individual residues within this motif.

As a TNF-related death factor, FasL is a type II transmembrane protein that shares roughly 25 to 30% sequence homology to other superfamily members such as tumour necrosis factor a (TNFα) [1]. Fig. 2 schematically depicts the FasL structure. The N-terminal region of the molecule harbours several putative phosphorylation sites which recently have been implicated in different aspects of signal transduction. Among those, FasL contains a binary casein kinase I (CKI) substrate motif that is similar to motifs in other TNF family members and, in the case of TNF, was implicated in reverse signalling and the regulation of expression [27]. In contrast to all other members of the protein family, however, the intracellular portion of FasL comprises an extended proline-rich domain (PRD) that serves as a docking site for proline-interacting Src-homology 3 (SH3) or WW domains (named because of the presence of two tryptophan residues in a certain spacing) [17].

FasL presumably exhibits its biological activity as a homo-trimeric complex. This oligomerisation seems to be essentially dependent on the self assembly (SA) region spanning aa 137 to 183 of the FasL ectodomain. The following TNF homology domain (THD) expresses the highest similarity to related proteins, but also mediates highly specific binding to the cystein-rich domains of the Fas receptor [28,29]. Within its ectodomain, the FasL contains three putative sites for N-linked glycosylation (N184, N250, and N260) which might be posttranslationally modified under experimental conditions but do not seem to alter self-aggregation or receptor binding significantly. However, since mutations of these residues correlate with a reduced expression of FasL in transfectants [28], it was suggested that glycosylation positively affects FasL maturation and/or stability. Moreover, secretion of soluble FasL (see below) strictly depends on glycosylation in 293 transfectants [30]. Of note, extensive N-linked glycosylation is often a characteristic feature of membrane proteins associated with lysosome function and appears to protect such proteins from degradation in the hydrolytic lysosomal environment [31]. Since FasL localises to secretory lysosomes in hematopoietic cells [32], N-glycosylation of the ectodomain might also have a comparable protective effect. How exactly these N-linked glycans enhance FasL expression in a given cell type remains to be addressed. So far, no crystallographic and nuclear magnetic resonance (NMR) spectroscopic data for FasL are available. However, in order to identify critical residues at the FasL-Fas interface, comparative molecular modelling with other TNF superfamily ligands revealed that this interaction is unaffected by the three FasL glycosylation sites [30,33,34].

In addition to glycosylation, other covalent posttranslational modifications including phosphorylation, methylation, acetylation and ubiquitylation represent powerful means of functional regulation. Complex patterns of such alterations are well documented for example for the tumour suppressor protein p53 [35]. In recent years, accumulating experimental evidence suggests that also the FasL might be subject to various regulatory modifications. Phosphorylation of the tyrosine residues Y7, Y9, and Y13 within the N-terminus of human FasL as well as ubiquitylation at lysine residues flanking the PRD, independently affect FasL sorting to secretory lysosomes [36]. A CKI-mediated serine phosphorylation within the murine FasL cytosolic tail following TCR engagement and stimulation with Fas-IgG fusion proteins was implicated in reverse signalling in CD8^+ ^T cells [26,37].

### Interactions via the FasL cytosolic PRD

Compared to other TNF family ligands, the cytoplasmic tail of FasL is unique with respect to its overall length (80 aa) and the presence of an extended polyproline stretch (aa 37 to 80). Other TNF superfamily ligands comprise much shorter N-terminal cytosolic stretches (human TNFα 35 aa, lymphotoxin β 18 aa and TRAIL 17 aa), and do not contain a PRD as present in the FasL N-terminus. As expected, this PRD allows for binding of SH3 domains with class I and II as well as non-canonical type I binding specificity and WW domains [38] and thus acts as a docking site for numerous proteins that exert significant impact on FasL biology and function (for a detailed review, refer to [17]).

An initial *in vitro *screen performed in 1995 used immobilised FasL-derived peptides and recombinant SH3 domains in order to analyse binding of such proteins to the FasL PRD [39]. Other strategies to identify interaction partners in T cells included pull down experiments with various recombinant SH3 domain fusion proteins or using recombinant human FasL-glutathione S-transferase (GST) fusion proteins followed by one- or two-dimensional gel electrophoresis [40,41]. A similar approach was recently undertaken in neuronal cells [42]. Moreover, some FasL associated factors or interacting proteins were identified by yeast two-hybrid library screening [43].

Among the identified SH3 domain proteins were several Src-related tyrosine kinases (Fyn, Lyn, Lck, Hck, Fgr, Src and Abl) as well as adapter proteins involved in T cell receptor-associated signal transduction (such as Grb2, Gads, the p85 subunit of the PI3 kinase and others) [36,39,40]. In addition, adapter proteins such as Nck, which for instance regulates changes in the actin cytoskeleton upon triggering of the TCR [44], and members of the Pombe Cdc15 homology (PCH) family were shown to bind to FasL and to affect its trafficking in hematopoietic cells [43-45]. In this context, it is noteworthy that PCH family proteins are getting more and more attention as important regulators of membrane dynamics and membrane-cytoskeleton interactions [46,47]. In the following, we focus on those proteins that have been mainly implicated in FasL reverse signalling and trafficking. For more general information about the FasL interacting SH3 and WW domain proteins identified so far, please refer to our recent reviews [16,17].

### Implications for FasL reverse signalling

Although the molecular details are still under investigation, reverse signalling via TNF ligands as a means to increase the plasticity of immune cell functions is meanwhile generally accepted [25]. In case of FasL, only recently several protein modifications and interacting molecules have been highlighted to play a role in the regulation of T cell activation [48]. The published results, however, are somehow contradictory in that different outcomes are seen in different T cell subpopulations.

**Figure 3:**
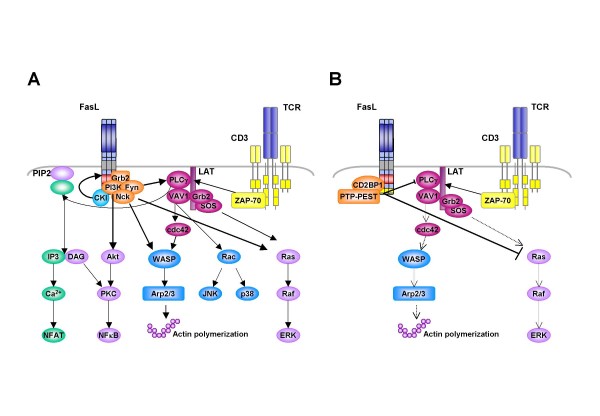
**Overview of FasL as a positive (A) or negative (B) costimulatory molecule in T cell activation**. Studies with murine CD8^+ ^T cells revealed a strong costimulatory capacity of FasL on signal transduction in TCR-stimulated cells, associated with enhanced MAPK, NFκB and PLCγ activation. In this scenario also the phosphorylation of FasL itself through the CKI as well as the enhanced WASP activation relevant for TCR clustering seems to be important (A). In contrast, FasL has been shown to completely block T cell activation (B). In human CD4^+ ^cells, cell cycle arrest appears to be caused by reduced WASP/Arp2/3-mediated TCR internalisation and inhibition of PLCγ and MAPK activation, possibly initiated through an interaction with the adaptor protein CD2BP1 and the tyrosine phosphatase PTP-PEST. In COS cells, ternary FasL-CD2BP1-PTP-PEST complexes have previously been observed [43].

In murine CD8^+ ^cells, FasL reverse signalling seems to provide a positive costimulatory signal required for maximal proliferation *in vitro *and *in vivo *[49,50]. The capacity to transduce such presumably costimulatory signals has been followed in more detail in the past years. Sun *et al*. suggested that cross-linking of the cytosolic portion of murine FasL was sufficient to positively costimulate non-transformed murine CD8^+ ^T cells following TCR engagement [26]. This was accompanied by FasL recruitment into lipid rafts, which is believed to allow formation of a signalling scaffold for the TCR-to-FasL crosstalk. They also observed a recruitment of Grb2, Fyn, and the p85 subunit of PI3K to the PRD and a serine phosphorylation of FasL. Costimulation resulted in activation of the MAPK pathway and eventually in the upregulation of IFN-γ production [26]. A possible model of a positive costimulatory signal initiated by FasL is depicted in Fig. 3A.

In their system, Fink and co-workers defined two independent motifs within the FasL cytoplasmic tail that seem to be crucial elements for reverse signalling [37]. The binary CKI substrate motif serves as target for the casein kinase resulting in serine/threonine phosphorylation of FasL, similar to what has been previously described for TNFα [27]. In addition, aa 45 to 50 of the PRD of murine FasL are also indispensable for costimulation, stressing the involvement of SH3 and WW domain proteins in this signalling process. Interestingly, although costimulation-associated SH3 domain proteins seem to bind to this short portion of the PRD, aa 45–50 do not seem to be required for FasL delivery to the cell surface. In essence, these reports indicate that the CKI recognition/substrate site and the PRD cooperate during FasL-mediated costimulation of CTLs. Of note, in this context, FasL reverse signalling strictly depends on CD3 activation but seems to be independent from classical costimulators such as CD28 and therefore presumably employs yet unidentified distinct signalling pathways [26,37].

In contrast to the costimulatory effect described above, Desbarats and colleagues detected a cell cycle arrest in primary murine CD4^+ ^cells upon FasL engagement [51]. Whether this discrepancy is due to the different degree of FasL crosslinking or indeed points to cell-type specific effects, is still unclear. Our own studies on the impact of FasL ligation on the TCR/CD3-induced activation of primary human T cells also revealed a complete block in proliferation of peripheral blood mononuclear cells, as well as isolated CD8^+ ^and CD4^+ ^T cells. This cell cycle inhibition is associated with a reduced expression of activation markers, decreased MAPK and PLCγ activation and a reduced IL2-production. Interestingly, also the activation-induced TCR internalisation is significantly inhibited by FasL ligation, indicating a role of FasL in the formation of essential signalling platforms [Paulsen *et al*., *Int. Immunol*., in revision]. Of note, the effects on human T cells in our study were reproducibly seen with plate-bound Fas-Fc alone, with Fas-Fc bound to anti-IgG-precoated plates, with oligomerised Fas-TNC-Fc fusion protein or anti-FasL polyclonal antibody, indicating that they are independent of the degree of cross-linking. Fig. 3B depicts the current hypotheses for negative costimulatory signals transduced via FasL.

### Regulation of FasL sorting and trafficking

As stated earlier, another important aspect of FasL biology is that its surface expression on T and NK cells has to be strictly regulated. This implies a safe intracellular storage and the potential for a rapid mobilisation, for example in a target cell contact situation. Therefore, in NK cells and CTLs, FasL is localised to so-called secretory lysosomes [32]. These specialised lysosomes are dual-function vesicles serving as degradative organelles comparable to conventional lysosomes, but also as storage compartment for secretory cargo proteins, including for example granzymes and perforin [52,53]. As also demonstrated in Fig. 1, upon activation, secretory lysosomes are released into the cytotoxic immunological synapse in a degranulation process referred to as "kiss of death" [5]. Unlike other lysosomal membrane proteins, however, FasL employs a unique sorting pathway independent of di-leucine or tyrosine-based sorting motifs [54]. Apparently, this pathway also depends on the presence of the FasL PRD. It was therefore speculated or assumed that SH3 domain proteins bind the FasL cytosolic PRD at the *trans*-Golgi network and then facilitate the transport of FasL-containing vesicles to the secretory lysosome [22]. Whereas deletion of the FasL PRD results in a prominent membrane association in hematopoietic cells, in non-hematopoietic cells, lacking these specialised organelles, FasL is constitutively localised to the cell surface [22].

Morphologically, secretory lysosomes might resemble multivesicular bodies (MVBs) with FasL potentially being a transmembrane component of the "inner vesicles". Thus, FasL released in association with microvesicles has also been observed [55,56]. Formation of MVBs has been extensively studied in the context of the epidermal growth factor receptor (EGFR) [57]. Upon induction by specific stimuli, e.g. ligand binding, the EGFR is phosphorylated as a prerequisite for its subsequent mono-ubiquitylation. In the lysosomal compartment, endosomal sorting complex required for transport (ESCRT) proteins bind this ubiquitin tag and assemble into a supramolecular complex facilitating the formation of lumenal vesicles. Zuccato and co-workers recently showed that FasL sorting to the MVBs was strictly dependent on ubiquitylation at positions K72/K73, two lysine residues close to the PRD, and on tyrosine phosphorylation at positions Y7, Y9, and Y13 in the human FasL. This phosphorylation was catalysed by Src-related kinases such as Fgr, Fyn, or Lyn, which were recruited to FasL via its PRD [36]. The tyrosine residues, however, are not conserved among species. Murine FasL, for instance, lacks Y9 and Y13 (Fig. 2) indicating that these three phosphorylated tyrosines in human FasL might not contribute equally to the sorting of FasL to MVBs. This, however, might also suggest that phosphorylation at residue Y7 is crucial for this process. Future experiments employing human FasL carrying point mutations at the individual tyrosine residues will clarify this issue. Interestingly, the potential phosphorylation sites are not conserved among other TNF ligand superfamily members, underscoring the unique nature of the FasL cytosolic portion and its capability to convey reverse signalling.

Some recent studies suggest that the intracellular storage compartment for FasL might in fact be distinct from the previously described secretory lysosomes [21,58]. Based on inspection by confocal microscopy, it appeared that in murine CTL clones and CD8^+ ^T and NK cell blasts, FasL does not or only partially colocalise with standard lysosomal markers including CD63 and Lamp-1 or the cytotoxic effector molecules granzyme B and perforin. Moreover, functional assays revealed that the requirements to elicit FasL or Lamp-1 surface appearance differ, indicating that indeed distinct storage compartments might be employed in the cells tested [21,58]. Hence, more experiments using biochemical and microscopical approaches are needed to definitely identify the FasL storage compartment and the mechanisms of FasL-associated lysosomal trafficking. Of note, proteomic analyses of enriched secretory granules from NK cells revealed major cell-line specific differences with respect to functionally relevant cargo proteins [59]. In this study, however, FasL and the aforementioned lysosomal marker proteins were clearly enriched in a single fraction during lysosomal purification. Similar observations were made when different T cell populations were investigated [Schmidt *et al*., personal communication]. This again suggests that secretory lysosomes/granules might be more versatile than initially anticipated, presumably depending on the type and the activation/maturation state of the cell they are originating from.

**Figure 4:**
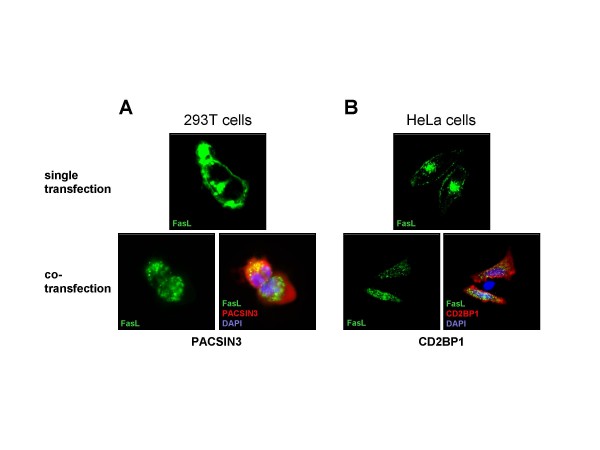
**PCH proteins regulate FasL expression**. If FasL-EGFP alone (upper panel) is transiently expressed in non-hematopoietic 293T and HeLa cells, a significant portion appears on the cell surface (and in a Golgi-like compartment). Upon co-expression of myc-tagged PACSIN3 or CD2BP1 (lower panel), FasL is predominantly located intracellularly and associates with distinct granular structures where it partially colocalises with the respective PCH protein. Cells were either transfected by calcium phosphate precipitation (A) or electroporation (B). 24 h after transfection, the cells were fixed, permeabilized and stained with anti-FasL mAb NOK-1, the anti-myc-tag mAb 9B11, corresponding AlexaFlour546-conjugated secondary antibodies and with DAPI to visualize nuclei.

Nevertheless, several other aspects of FasL trafficking have been associated with SH3 domain proteins that interact with the FasL PRD. We and others previously described members of the PCH protein family, including FBP17, the PACSINs 1–3, CD2BP1/PSTPIP and CIP4 as putative FasL interactors [42,43,45]. As mentioned, if overexpressed in non-hematopoietic cells, FasL is localised to the cell surface [22]. Cotransfection with PCH proteins, however, results in an intracellular retention of FasL, colocalisation with components of the lysosomal compartment [43,45] and reduced killing capacity of transfected 293T cells [43] (Fig. 4). PCH proteins could be particularly important interactors of FasL, since they are known to govern the cross-talk between membrane and cytoskeleton dynamics [46,47]. Therefore, PCH proteins are likely to be relevant for the trafficking of FasL-containing vesicles (discussed in more detail in [17]). In this context, we recently screened for protein interactions of FasL-binding PCH proteins in T cells [60]. In essence, our data support a scenario in which PCH proteins might directly link FasL-loaded vesicles to the Wiskott-Aldrich syndrome protein (WASP) and the WASP-interacting protein (WIP) which in turn serve as key regulators for the subsequent Arp2/3-dependent actin polymerisation.

We also showed that FasL interacts with the classical adapter protein Nck that is ubiquitously expressed and contains three SH3 domains and one SH2 domain [40]. It is known that Nck links TCR-associated tyrosine kinases to regulative components of the actin cytoskeleton, for instance WASP and the Arp2/3 complex [[61], and Lettau *et al*., CCS in revision]. In functional terms, we demonstrated that Nck is essential for the transport of FasL-carrying (secretory) vesicles to the cytotoxic immunological synapse [44]. Therefore, Nck links TCR signalling in a tyrosine kinase- and actin-dependent manner to the transport of these cytotoxic vesicles. Also in neuronal cells, FasL interactors have been implicated in the regulation of its surface expression. However, in Schwann cells, the adapter protein Grb2 seems to link FasL to adaptin β and thereby promotes sorting of FasL to the cell surface by a potentially different mechanism [62].

### Regulation of FasL cytotoxicity

So far, we described posttranslational modifications of FasL linked to either its trafficking and activation-induced release or its capacity to transduce reverse signals. However, we also know that several other mechanisms of posttranslational modification or molecular positioning may influence the function of FasL. These include raft association and proteolytic processing.

Lipid rafts are membrane microdomains enriched in (glyco-)sphingolipids and cholesterol. This local microenvironment is believed to allow tight packaging of lipids as well as integral membrane proteins, thus forming scaffolds for instance for signal transduction processes [63]. However, although lipid rafts provide explanations for several transmembrane signalling processes, only recently their existence as well as their dynamics and exact involvement in membrane-associated biological processes have been questioned. Nonetheless, two studies provide microscopic and biochemical evidence as well as fluorescence correlation spectroscopy data showing that overexpressed as well as endogenous FasL partially localises to lipid rafts. Moreover, binding to its receptor Fas results in an increased recruitment of FasL to these membrane microdomains [64] and chemical disruption of rafts reduces the cytotoxic potential of the cells tested [64,65]. These data are consistent with prior observations that FasL forms durable supramolecular clusters [66] and that FasL is concentrated in lipid rafts in a cytoskeleton-dependent manner [67]. Again, the importance of the FasL PRD was underscored also in this context, since recruitment into raft areas strictly depends on the presence of this domain [65]. It was even assumed that lipid raft localisation is required for clustering and aggregation of FasL [65]. This in turn seems to be prerequisite for effective Fas signal induction in target cells [68].

Since many regulated properties of FasL apparently depend on interactions of SH3 domain containing proteins with the PRD, it was surprising that Jodo and colleagues reported that the very N-terminus of FasL – aa 2 to 33, excluding the PRD – were crucial to convey its cytotoxic property [69]. Deletion of this portion resulted in 30- to 100-fold reduced cytotoxic potential suggesting a regulation across the membrane barrier. In their study, however, they used an artificial system employing FasL microvesicle preparations. Thus, the functional consequences of an N-terminal deletion sparing the PRD remain to be elucidated in living cells.

### Proteolytic processing of FasL

Proteolytic processing by ectodomain shedding and regulated intramembrane proteolysis (RIP) is a common feature of many different membrane proteins. TNFα was one of the first molecules for which shedding was described in more detail. This eventually led to the initial cloning of ADAM17, a key metalloprotease responsible for ectodomain shedding of various membrane proteins [70].

In the meantime, shedding of transmembrane substrate proteins is in many cases attributed to the Zn^2+^-dependent proteolytic activity of members of the a disintegrin and metalloprotease (ADAM) family of metalloproteases [71]. Substrates for ADAM metalloproteases include cell adhesion molecules, transmembrane chemokines and cytokines, cell surface receptors and others. Various aspects of ADAM-mediated shedding have been reviewed recently [71-73], particularly its importance in cell-cell adhesion [74], leukocyte transmigration [75], EGFR signalling [76] and in immunity in general [77]. Shedding not only generates soluble ectodomains or fragments, but also leaves membrane-embedded remnants referred to as C-terminal or N-terminal fragments (CTFs and NTFs, respectively) depending on the substrate topology. As shown for numerous substrates, these CTFs or NTFs may then be subject to further proteolytic processing (RIPping) by intramembrane-cleaving proteases (I-CLiPs) [78]. I-CLiPs are multipass membrane proteins and include for instance the presenilins that have been implicated in the pathology of Alzheimer's disease [79]. I-CLiP activity generates soluble intracellular fragments of substrate proteins which may have several potential fates including the regulation of gene activity. As an example, shedding and subsequent RIPping of Notch regulates T and B cell development. In this case, the γ-secretase complex releases an intracellular domain into the cytosol which subsequently translocates to the nucleus and modulates transcriptional activity of developmentally relevant genes [77,80]. Similarly, the shedding of cadherins releases β-catenin from membrane-associated complexes into the cytosol and nucleus, thereby also regulating transcription [81,82].

Interestingly, very soon following the initial characterisation of FasL, a soluble fragment of FasL (sFasL) of approximately 26 kDa was described to be generated by metalloprotease-mediated cleavage or alternate splicing [83-85]. Since then, sFasL has been implicated in various pathological conditions. Different cleavage sites of human FasL were mapped by Edman degradation of purified sFasL depending on the cellular context (see Fig. 2). Of note, sFasL was shown to possess a significantly lower cytotoxicity compared to membrane FasL (mFasL) [86,87]. Initially, matrix metalloprotease 7 (MMP7) emerged as candidate FasL sheddase, since it was described to cleave FasL in certain cell types. However, in contrast to the previously described sFasL fragments, the soluble protein generated by MMP7 cleavage had pro-apoptotic activity, associated with development of pancreatic metaplasia [88]. Therefore, FasL might in fact be processed differentially in a cell-type dependent manner. This could also explain why several potential MMP7 cleavage sites have been described within murine and human FasL [89]. Again, the exact nature of sFasL generated by ectodomain shedding and its pro- or anti-apoptotic function appears to depend on the cellular microenvironment and also on its interactions with components of the extracellular matrix [90].

Since the close FasL-relative TNFα is cleaved primarily by ADAM17 [70] and given that ADAM proteases have been implicated in ectodomain release of various other substrates [71,73], their involvement in FasL shedding was suspected. Consequently, using ADAM-deficient cell lines, RNAi knockdown as well as ADAM-specific hydroxamate-based inhibitors, ADAM10 was identified as the major FasL sheddase in T cells [23,24]. In human T cells, ADAM10-dependent shedding of FasL also affected AICD in a superantigen stimulation model [23], suggesting that shedding might play a significant role in the regulation of FasL-mediated cytotoxicity. Interestingly, Kirkin and co-workers observed an 11 kDa FasL intracellular domain, which was generated via sequential proteolysis by ADAM10 and SPPL2a, an I-CLiP exhibiting specificity for type II membrane proteins. They also described that the ICD subsequently translocates to the nucleus and modulates reporter gene transcription [24]. Quite surprisingly, recent data suggest a similar scenario also for TNFα. Initially, a 9 to 11 kDa fragment of TNFα was observed in Western analyses of transfected HeLa cells. It was particularly enriched in nuclear fractions, consistent with a functional nuclear localisation signal in this N-terminal region [91]. More recent studies, however, detected a 6 kDa ICD generated by SPPL2a- and SPPL2b-mediated processing of an approximately 15 kDa TNFα NTF [92,93]. Moreover, SPPL2a/2b-mediated RIPping of TNFα was shown to trigger interleukin-12 release in dendritic cells and might thus be relevant for Th1 differentiation [93]. The TNFα ICD, however, lacks the characteristic PRD and the potential tyrosine phosphorylation sites present in the FasL N-terminus. Therefore, it is presently not clear how exactly the TNFa ICD is capable of triggering the observed effects.

In general terms, the proteolytic processing of FasL by shedding and RIPping provides a new level for the regulation of cell-mediated cytotoxicity and potentially for the regulation of intracellular (reverse) signal transduction. Therefore, it will be of particular importance to further define the mechanisms govern ADAM-mediated cleavage of FasL. Of note, the regulation of ADAM proteases with respect to substrate specificity and catalytic activity is still poorly defined [72]. Interestingly, however, several ADAM family members contain potential SH3 domain binding sites and some have been shown to interact with SH3 domain containing proteins, including adaptor proteins required for ADAM trafficking and localisation. As an example, PACSIN3, one of the PCH family proteins which also bind to the FasL, was associated with upregulation of ADAM12 activity and also seems to interact with the cytosolic tail of ADAM10 [94]. In addition, various kinases might act as regulators of ADAM activity [71].

**Figure 5:**
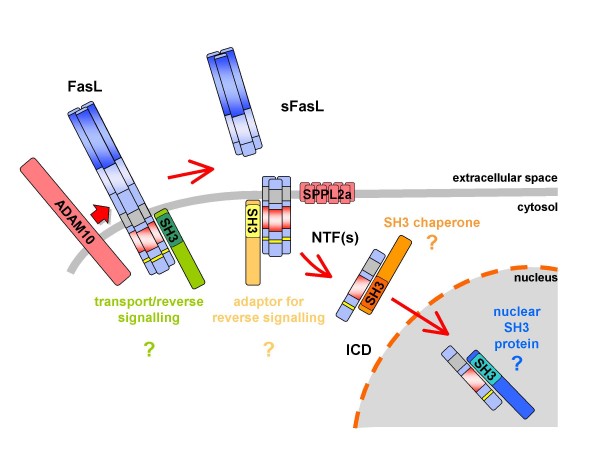
**FasL proteolytic processing**. Recent data suggest that FasL is processed by ADAM10 and RIPped by SPPL2a generating a free intracellular ICD. This ICD is assumed to translocate to the nucleus and to regulate gene expression, a process resembling the well characterised Notch signalling pathway. Since FasL is known to associate with SH3 domain proteins and since these interactions significantly affect FasL biology, including sorting, activation-induced release and reverse signalling, further studies have to determine how the processing of FasL affects interactions with SH3 domain proteins. Furthermore, it is unclear to date, which interactions regulate the trafficking of processed FasL fragments.

Apparently, more work is needed to address the exact fate of the FasL ICD generated by SPPL2a processing. Apart from its capacity to regulate transcriptional activity [24], the release of the FasL fragment including its CKI motifs, the polyproline stretch and its potential phosphorylation sites might also contribute other mechanisms for reverse signalling. It is still open, whether SH3 and WW domain proteins interacting with the cytosolic portion of full length mFasL would also bind to processed cytosolic (and potentially nuclear) FasL fragments. Likewise, it is not known whether and how such interactors affect the function and/or subcellular trafficking of FasL fragments (Fig. 5). Moreover, it has to be elucidated whether the FasL ICD itself is subject to processing, for instance by cytosolic pro-protein convertases, or whether it becomes modified by phosphorylation, ubiquitylation or similar mechanisms. Of note, FasL proteolysis is not necessarily restricted to metalloprotease activities. A recent study reported that murine FasL might also be cleaved at positions R144-L145 by plasmin, releasing a pro-apoptotic extracellular fragment from endothelial cells [95]. Inhibition of this process by plasminogen activator inhibitor-1 favours angiogenesis in a mouse tumour model.

## Conclusion

FasL is a prototypic death factor and plays an essential role in immune cell effector functions as well as immune homeostasis. In spite of the fact that other functions than apoptosis induction have recently been elucidated for the FasL/Fas system, the FasL itself is potentially dangerous and requires a strict control which is achieved at several levels by posttranslational mechanisms. Clearly, the unique polyproline stretch plays a major role in FasL biology. SH3 (and WW) domain interaction partners have emerged as potent regulators of FasL sorting, storage, trafficking and activation-induced release. Nonetheless, there is also increasing evidence that both tyrosine and serine/threonine phosphorylation in the FasL cytosolic tail might contribute to its function as a death inducer and a retrograde signal modifier.

The observation of FasL RIPping by SPPL2a and the generation of a fragment that is presumably capable to translocate to the nucleus to regulate gene transcription, opened a completely new chapter of FasL biology. Especially in view of the numerous potential SH3 domain interactions, future work should reveal whether processed fragments are also capable of interacting with the same or a different set of SH3 domain proteins and address the functional consequences of these interactions.

## Competing interests

The authors declare that they have no competing interests.

## Authors' contributions

MV designed and wrote the initial version of the article in close collaboration with ML who also provided original data presented as figures. Based on her own experimental work, MP contributed significantly to the discussion of reverse signalling of FasL. OJ supervised the work and holds responsibility as the corresponding author. All authors have read and approved the final manuscript.
